# A case report of a concomitant total thyroidectomy and carotid body tumor resection in a 43 year old female

**DOI:** 10.1016/j.ijscr.2018.10.019

**Published:** 2018-10-17

**Authors:** Ramon Garcia-Alva, Luis O. Bobadilla-Rosado, Luis H. Arzola, Monserrat Escobar-Preciado, Javier E. Anaya-Ayala, Carlos A. Hinojosa

**Affiliations:** Department of Surgery, Section of Vascular Surgery and Endovascular Therapy, Instituto Nacional de Ciencias Médicas y Nutricion Salvador Zubirán, Mexico City, Mexico

**Keywords:** Thyroidectomy, Carotid body tumor, Goiter

## Abstract

**Introduction:**

Carotid body tumors (CBT) are rare neoplasms with relatively; due to their malignant potential, the indicated treatment is surgical resection. There have been described some associations of carotid body tumors with other neoplasms, however, the concomitant presentation of CBT with multinodular goiter is rarely encountered.

**Presentation of case:**

We herein present the case of a 43-year old woman with the aforementioned association. As a part of the diagnostic evaluation, an ultrasound was performed, revealing both conditions simultaneously. The surgery took place with the aim of resecting both lesions during the same intervention. The patient was discharged without complications.

**Discussion:**

The concomitant presentation of goiter and carotid body tumor is rarely reported but an extended Kocher incision could be enough to an adequate exposure and same surgical procedure resection.

**Conclusion:**

This case reinforce the concomitant assessment in two different pathologies. Although there is one case reported before, this case lead to improvement in the treatment of these patients.

## Highlights

• Carotid body tumors (CBT) are rare neoplasms with malignant potential.
• The concomitant presentation of a CBT with goiter has been only reported in one case in 1950.
• The extended Kocher incision for resection of both tumors was performed succesfully.

## Introduction

Carotid body tumors are rare neuroendocrine tumors originating from the sympathoadrenal and parasympathetic paraganglia of the autonomic nervous system. The incidence of this tumors is estimated to be of 1:30,000 to 100,000 in the general population [1].

Thyroid nodules represent a common clinical problem, nonetheless only 5% of them are palpable, while incidental diagnosis with a cervical ultrasound is thought to be over 70%, with a greater prevalence in elderly patients [2]. While malignancy can be a possibility in these nodules, benign follicular nodules either solitary or as part of a multinodular goiter have been reported to be the most common type [2]. Multinodular goiter is not a rare entity (it presents endemically in 5% and sporadically <5% of population) that is characterized by the presence of multiple functioning or non-functioning thyroid nodules [3,4].

The association of multinodular goiter with carotid body tumors or paraganglioma, is a rare form of presentation; with very few cases reported in the literature [[5], [6], [7], [8], [9]]. The treatment for carotid body tumors has long been known to be surgical resection. Multinodular goiters may be treated with surgery, suppressive therapy with thyroid hormones plus iodine or radioiodine according to different regions but there is general agreement that medical treatment is ineffective for substernal goiters; which makes surgery the best choice of treatment [[10], [11], [12], [13], [14]]. We present the case of an intrathoracic multinodular goiter associated with carotid body tumor (Shamblin II) that were treated with surgical resection during the same surgical time. This case study is constructed in order to satisfy the Scare criteria [15].

## Case presentation

**Fig. 1:**
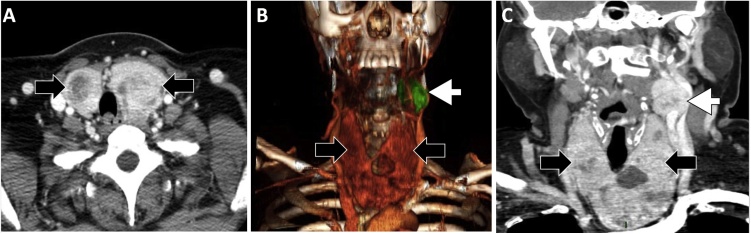
Goiter (black arrows) and paraganglioma (white arrows) in a computerized tomography angiography (CTA). A) Axial view demonstrates displacement of the airway to the right, B) Three-dimensional reconstruction, C) Coronal view.

A 43-year-old woman was referred to our institution with a neck mass in the left submandibular region. She was studied in an outside hospital, where a neck ultrasound showed a multinodular goiter and a CBT on the left carotid bifurcation. A neck Computed tomography angiography (CTA) demonstrated a carotid body tumor (3.8 cm × 2.5 cm × 3.3 cm) classified as Shamblin II and an intrathoracic multinodular thyroid gland with a right lobe extension of 11.9 cm × 9.7 cm and the left lobe of 25.2 cm × 21.3 cm with caudal retrosternal growth and evidence of slightly trachea deviation (Fig. 1).

**Fig. 2:**
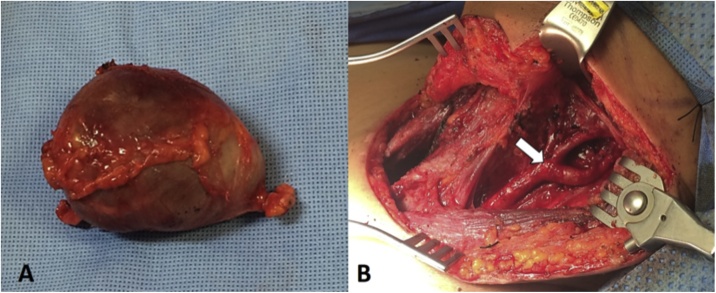
A) Carotid body tumor piece B) Extended Kocher incision (white arrow shows the carotid bifurcation).

**Fig. 3:**
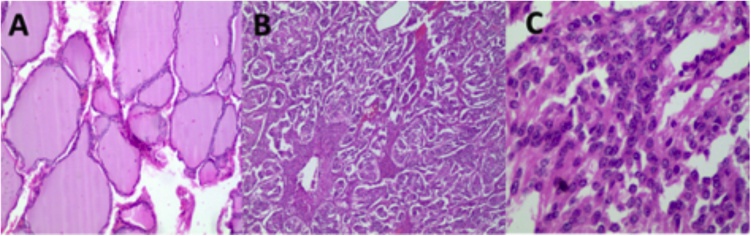
A) Thyroid follicles filled with colloids with variability in their size. (B–C glomus), B) Tumor nests separated by a fibrovascular septum (Zellballen), C) Eosinophilic cells with indistinct edges, round nucleus and chromatin with “salt and pepper” appearance.

Thyroid function test (TFT) were normal, serum antithyroglobulin antibodies and thyroperoxidase antibody (TPO) were undetectable. Thyroid gammagram was performed reporting a multinodular goiter. Fine needle aspiration was performed demonstrating nonmalignant cells. A total thyroidectomy was performed with a transverse lower neck incision (Kocher incision), posteriorly, CBT was resected by an extension of the previous Kocher incision to the anterior border of the sternocleidomastoid muscle using the retrocarotid reported previously in our group as an effective technique, also two surrounding lymph nodes were resected to rule out malignancy (Fig. 2). The pathology report demonstrated a paraganglioma with negative lymph nodes invasion and a multinodular goiter (Fig. 3).

The patient recovered satisfactorily after the surgery and she was discharged in postoperative day number three, she currently continues her follow-up as our patient. Twelve months after the surgery, she does not present any complaints. She is currently on levothyroxine and calcium carbonate.

## Discussion

Neck lymphadenopathy or tumors evaluation require a careful medical history and a thorough physical examination of the patient. Multiple causes of volume increase in the neck have been reported previously; within most common causes, we could divide into 4 different groups: 1) infections (mycobacterial, viral, fungal), 2) malignancies (hematologic and solid tumors), 3) immunologic disorders (connective tissue disorders, serum sickness, and sarcoidosis) and 4) miscellaneous (Lymph node hyperplasia) [23]. Multinodular goiter represents one of the solid tumor type adenopathy that should be ruled out because it is common form of presentation.

Most patients with multinodular goiter have few or no symptoms except for those with large goiters. Many patients are referred to the hospital for cosmetic reasons or, more frequently, for compression of cervical structures causing tracheal deviation, shortness of breath, cough, voice changes, odynophagia and dysphagia [3,16]. While there is still controversy on how a substernal goiter is defined, there are some criteria that have been used such as a thyroid gland extending 3 cm below the sternal notch or below the 4th thoracic vertebrae [17]. When it comes to treatment, the presence of a substernal goiter is enough to have an indication for surgical management [17,18], however, controversy still exists on which type of surgical treatment is considered the best option. Albayrak and colleagues recommend the use of a total thyroidectomy due to the possibility of complications in recurrent surgeries and the malignant potential of the thyroid tissue [16]. Hence, surgical treatment has shown a resolution of compressive symptoms caused by thyroid disease [16]. In the case herein presented a total thyroidectomy was used with good results and no complications during the perioperative.

On the other hand, the clinical presentation of carotid body tumors differs depending on their size. Carotid body tumors classically present as asymptomatic enlarging lateral neck masses [19] and most of the time, the patients only complaint of a neck mass. Carotid body tumors, also known as Head and Neck Paragangliomas (HNP) that are located at a lower level, may be confused with goiters which can cause an under diagnosis especially due to the rarity of these tumors. Carotid body tumors are classified anatomically according to Shamblin et al. [20] and their surgical treatment concise of a total resection for which the Standard Caudocranial Technique (SCCD) is widely used. In our hospital, we utilized the surgical technique described previously by the senior author of this paper Hinojosa et al, named “Retrocarotid dissection”, which was introduced in our institution in 2007 and showed, a significant decrease in intraoperative bleeding and procedural time in comparison to the SCCD [21]. Compared to the case reported by Corbett and colleagues in 1950 [22] where both tumors were resected by separate incisions; we decided to perform an extended Kocher incision (a longitudinal incision along the anterior border of the sternocleidomastoid) with an appropriate exposure of the thyroid gland and the carotid bifurcation (Fig. 2B), this extended incision allowed adequate exposure of the surgical field, providing better cosmesis for the patient instead of performing a second incision for the carotid body tumor resection. To the best of our knowledge, the association of carotid body tumor with multinodular goiter has only been described once in a 60-year-old male with both of the masses being asymptomatic [22].

## Conclusion

In conclusion, the association of paraganglioma with multinodular goiter is rare and has a good prognosis according to few cases reported in the literature. Surgery is the treatment of choice, and resection of both lesions in a single procedure is feasible in most cases without increasing morbidity. In this particular case, a left extended cervical incision allowed successful removal of both tumors without any complication.

## Conflicts of interest

The authors have no conflict of interests.

## Funding

No sponsors involved

## Ethical approval

This case is exempt from institutional ethnical approval

## Consent

Written informed consent was obtained from the patient for publication of this case report and accompanying images. A copy of the written consent is available for review by the Editor-in-Chief of this journal on request".

## Author contribution

Ramon Garcia-Alva, Data collection, writing the manuscript

Luis Bobadilla Writing the manuscript.

Luis H Arzola Writing the manuscript, data collection.

Monserrat Escobar-Preciado, Data collection.

Javier E. Anaya-Ayala, Critical review of the article.

Carlos A. Hinojosa Conception and design. Critical review of the article.

## Registration of research studies

Not required

## Guarantor

Dr. Carlos A. Hinojosa,

Professor of Vascular Surgery

## Provenance and peer review

Not commissioned, externally peer reviewed.
